# Lupus Nephritis With Mild Asymptomatic Hypercalcemia in Children: A Case Report and Literature Review

**DOI:** 10.3389/fped.2019.00507

**Published:** 2019-12-06

**Authors:** Lingling Xu, Jun Zhang, Ronghui Pu, Zhihui Yue, Ying Mo, Xiaoyun Jiang

**Affiliations:** Department of Pediatrics, The First Affiliated Hospital, Sun Yat-sen University, Guangzhou, China

**Keywords:** lupus nephritis, systemic lupus erythematosus, hypercalcemia, children, calcium sensing receptor

## Abstract

Lupus nephritis (LN) in Children is caused by Kidney involvement in systemic lupus erythematosus (SLE). SLE is a chronic, multi-systemic, paroxysmal autoimmune disease with a wide spectrum of autoantibodies. Hypercalcemia is a common manifestation of primary hyperparathyroidism and other malignant tumors. LN or SLE with hypercalcemia is rare. We report a case of LN with hypercalcemia and review the literature. It suggests that the pathogenesis of SLE complicated with hypercalcemia complicates the response of drug therapy.

## Background

Hypercalcemia is a relatively common clinical problem. The most common causes of hypercalcemia, affecting 90% of all patients, are primary hyperparathyroidism and malignancy. Lupus nephritis (LN) is caused by Kidney involvement in systemic lupus erythematosus (SLE). LN with hypercalcemia is rare, especially in children. To our knowledge only 17 cases of patients with SLE or LN with hypercalcemia, have been previously described. We present a case of SLE with hypercalcemia and review the literature.

## Patient Presentation

We report an 11-year-old male patient who was hospitalized on January 23rd, 2019 and found to have elevated serum calcium for 4 months. Two years ago (December 2016), due to proteinuria, hypocomplementemia, anti-nuclear antibody, and positive anti-dsDNA, he was diagnosed as SLE and LN. The treatment of high dose methylprednisolone plus high dose cyclophosphamide and plasma exchange did not improve his condition. Urinary protein continued to be 4+~5+. Then mycophenolate mofetil (MMF) plus tacrolimus (FK 506) were used to treat. Glucocorticoid was gradually decreased during this period. MMF (0.375 g q12h, concentration of MMF is 53.741 ug/ml), FK 506 (1.5 mg qm and 1.0 mg qn, concentration of FK506 is 4.8 ug/L), and prednisone (10 mg qd) were being taken to treat LN. He had no history of taking excessive calcium and vitamin D preparations. There was no clinical manifestation of hypercalcemia at admission. At admission, SLE was in remission, urinary protein was negative, and SLEDAI score was 2 points (positive for anti-dsDNA). Physical examination: height 144 cm, weight 47.5 kg, body surface area 1.33 m^2^, no edema, superficial lymph node swelling, cardiopulmonary abdominal examination no abnormalities.

Laboratory examination at admission: hypercalcemia (2.88–3.09 mmol/L, normal range 2.1–2.6), low blood phosphorus (1.16 mmol/L, normal range 1.29–1.94), high iPTH (110.4 pg/mL, normal range 12–88), 25-hydroxyvitamin D deficiency (11 ng/mL, normal range > 25), normal alkaline phosphatase (224 U/L, normal range 0–110), magnesium (0.64 mmol/L, normal range 0.7–1.0), Leukocyte count was 11.54 × 10^9^/L (normal range 4.0-10.0), hemoglobin was 14.6 g/dL (normal range 12.0–15.0), and platelet count was 444 × 10^9^/L (normal range 100–300); blood Urea Nitrogen (BUN) was 6.1 mmol/L (normal, 2.9–8.6), creatinine (CR) was 80 umol/L (normal, 53–115), creatinine clearance rate (CCR) 129.04 ml/min.1.73 m^2^. Thyroid function is normal. The level of anti-dsDNA was 14.01 IU/mL (normal range 0–12), and the anti-nuclear antibody was normal. Blood complement C3 and C4 were normal. Renal biopsy: immunocomplex nephritis, lupus nephritis, segmental mesangial proliferation, mild activity, LN III(A/C).

Erythrocyte sedimentation rate was normal. Urinary protein was negative. Twenty four hours urinary calcium was 4.07 mmol (normal range 2.5–7.5). His electrocardiogram was normal. Chest X-ray showed no abnormality in cardiopulmonary septum. CT scan of parathyroid imaging showed that SPECT/CT imaging was performed 15 and 120 min after intravenous injection of 99mTc-MIBI; 15 min showed that the left lobe of thyroid was irregular in shape; Radioactive aggregation could be seen at the lower pole of left lobe, and the right lobe of thyroid was normal; 120 min showed that the bilateral thyroid became pale obviously, and radioactivity could still be seen at the lower pole of the original left lobe. CT tomography and fusion images showed uniform low-density abnormal shadow of about 8 mm ^*^7 mm in size below the left thyroid lobe, about the level of the first thoracic vertebra, with relatively uniform internal density, no obvious calcification, CT value of about 34 HU, slight abnormal 99mTc-MIBI concentration in the corresponding part of the fusion image; parathyroid imaging (posterior lower left lobe) Yang Sex. SPECT/CT indicated hyperplasia of the parathyroid gland. Ultrasound showed hyperplasia of the parathyroid gland. Hypercalcemia caused by malignant tumors has been excluded during hospitalization.

## Treatment

At the beginning of hypercalcemia treatment, the low calcium diet, intravenous saline infusion and furosemide were given to increase calcium excretion. Continue to treat LN with MMF, FK 506, and prednisone, the decrease of serum calcium was not obvious, and after hydrotherapy and diuretic treatment, the decrease of serum calcium was not obvious, the fluctuation of serum calcium was 2.88–3.09 mmol/L, and there was a stage 1 of acute kidney injury (2019-2-2 BUN 8.9 mmol/L, CR 87 umol/L, CCR 80.44 ml/min.1.73 m^2^). Parents refused hydrotherapy and diuretic treatment. The patient was discharged without any symptoms. CR decreased to normal (61 umol/L) 2 days after discharge. Parents refused to do genetic testing for multiple endocrine adenomatosis type 1. A half year follow-up: the patient is in good condition, without any medication. Under the control of the low calcium diet, his serum calcium level fluctuated at 2.7–3.0 mmol/L.

## Literature Review and Discussion

Hypercalcemia is a common electrolyte disorder. More than 90% of hypercalcemia cases are caused by primary hyperparathyroidism or malignant tumors. When primary hyperparathyroidism occurs, serum iPTH levels increase markedly, usually with parathyroid adenoma or hyperplasia. Malignant tumors include hematological and solid tumors.

We used the search terms “systemic lupus erythematosus” and “hypercalcemia” on PubMed to get 38 articles. The title and abstract were reviewed, and 19 unrelated articles and 1 comment were excluded. All the articles are case reports. After carefully reading and reviewing their references, 17 cases were finally confirmed (1–17). All cases had no history of taking excessive calcium and vitamin D preparations. Among 17 cases reported, there were 15 females and 2 males (7, 15); 1 mild hypercalcemia (7) (male), 5 moderate hypercalcemia (2, 8, 12, 15, 16) (1 male and 4 females), 11 severe hypercalcemia (1, 3–6, 9–11, 13, 14, 17) (all females); 2 children (all females) (6, 9), 15 adults; there were 14 cases of hypercalcemia in active stage of SLE, 3 cases of hypercalcemia in remission stage of SLE (3, 6, 12) (all female, 2 of them considered the main cause of non-hypercalcemia in SLE); 15 cases of SLE-related hypercalcemia (1, 2, 4–11, 13–17), and 1 case of hypercalcemia were caused by primary hyperthyroidism (3), The coexistence of SLE and hypercalcemia was considered in only 1 case (3); SLE with PTH elevation in 5 cases [including 3 cases of parathyroid adenoma (1, 6, 12), 1 case of parathyroid cyst (3), 1 case was caused by autoantibody of calcium sensitive receptor (9)], SLE with PTHrP elevation in 3 cases (2, 15, 17); 1 case may be caused by false negative PTHrP (11), 2 cases may be caused by anti-PTHrP (4, 8), 5 cases may be caused by PTH-related protein and autoantibodies (7, 10, 13, 14, 16), 1 case may be related to the decrease of fibroblast growth factor 23 (5); 10 cases had increased serum creatinine or decreased creatinine clearance (2, 4–7, 9–11, 13, 15), 5 cases had LN (7, 9, 10, 13, 15); 7 cases had SLE variants, which were described as hypercalcemia-lymphedema Syndrome, characterized by hypercalcemia and serositis (4, 8, 13–17). Among them, 1 case was positive for PTHrP by lymph node biopsy (15); 6 cases were complicated with ectopic calcification (2–4, 9, 10, 13, 17). There were 1 case of SLE complicated with tumors (12); 2 cases died, one died of hypercalcemia crisis (1), the other died of refractory septic shock (9); 11 cases of hypercalcemia were effective by glucocorticoid therapy (2, 4, 5, 7, 8, 10, 11, 13–15, 17), 2 cases were ineffective by glucocorticoid therapy (1, 16); after surgical excision of parathyroid gland, blood calcium returned to normal in 3 cases (3, 6, 12) (Table 1).

**Table 1:** Literature review of SLE or LN with hypercalcemia.

**References**	**Age at diagnosis/** **year**	**Gender M/F**	**SLE0 related** **HCA**	**Degree** **of HCA**	**LA**	**LE**	**LN**	**RI**	**PTHrP** **(serum)**	**iPTH** **(serum)**	**Phosphate** **(serum)**	**25(OH)2-VitD**	**SLE** **activity**	**Comments**	**Ectopic** **calcinosis**	**Response to** **steroids**	**Parathyroid adenoma/** **cyst/** **hyperplasia**	**Surgical removal** **of parathyroid gland**	**Saline** **hydration**	**Calcitonin**	**Bisphosphonates**
Kiper et al. (1)^#^	77	F	Yes	Severe	Yes	No	No	No	UK	↑↑	↓	UK	Yes	Hypercalcemia May be associated with PTHrP, PTH autoantibodies, proinflammatory cytokines, and unknown individual factors	No	No	Yes	No	Yes	No	Yes
Zhao et al. (2)	39	F	Yes	Moderate	No	No	No	Yes	↑↑	↓	↑	Normal	Yes	Elevated PTHrP should be the main cause of hypercalcemia	Lung, the subcutaneous tissue of both hands	Yes	No	No	Yes	Yes	Yes
Jiang et al. (3)	62	F	No	Severe	No	No	No	UK	UK	↑↑	Normal	UK	No	Parathyroid cyst should be the main cause of hypercalcemia	Left kidney	UK	Yes	Yes	No	Yes	Yes
Abdul et al. (4)	27	F	Yes	Severe	Yes	No	No	Yes	UK	↓	Normal	UK	Yes	Hypercalcemia May be associated with PTHrP, PTH autoantibodies, and proinflammatory cytokines	Corneal	Yes	No	No	Yes	No	Yes
Zhang et al. (5)	76	F	Yes	Severe	No	No	No	Yes	Normal	↓	UK	Normal	Yes	FGF23 might be involved in the pathogenesis of hypercalcemia	No	Yes	No	No	No	No	No
Choudhry et al. (6)	13	F	Yes	Severe	No	No	UK	Yes	UK	↑↑	↓	↓	No	Hypercalcemia Was attributed to SLE, because she had low bone density	No	UK	Yes	Yes	Yes	Yes	Yes
Del et al. (7)	65	M	Yes	Mild	No	No	Yes	Yes	Normal	↓	Normal	Normal	Yes	Hypercalcemia May be associated with PTHrP, PTH autoantibodies, and proinflammatory cytokines	No	Yes	No	No	No	No	Yes
Karageorgas et al. (8)	29	F	Yes	Moderate	Yes	No	No	No	UK	↓	Normal	↓	Yes	Hypercalcemia May be associated with PTHrP, PTH autoantibodies, and proinflammatory cytokines	No	Yes	No	No	Yes	No	Yes
Kohli et al. (9)^#^	8	F	Yes	Severe	UK	No	Yes	Yes	UK	↑	↓	UK	Yes	Circulating autoantibodies to the extracellular Calcium sensing receptors were hypothesized determining hypercalcemia and elevated levels of PTH	Kidney	UK	No	No	No	Yes	Yes
Farah et al. (10)	19	F	Yes	Severe	UK	No	Yes	Yes	Normal	Normal	↑	Normal	Yes	Hypercalcemia May be associated with PTH autoantibodies	Lung	Yes	No	No	Yes	Yes	Yes
Erdozain et al. (11)	39	F	Yes	Severe	UK	No	No	Yes	Normal	↓	Normal	↓	Yes	The possibility of false negative PTHrP results from immunoradiometric assay is also stressed	No	Yes	No	No	Yes	No	Yes
Galrao et al. (12)^*^	47	F	No	Moderate	No	No	No	No	UK	↑↑	↓	↓	No	The relationship between SLE and hyperparathyroidism is unclear	No	UK	Yes	Yes	No	Yes	Yes
Berar-Yanay et al. (13)	48	F	Yes	Severe	Yes	Yes	Yes	Yes	Normal	↓	Normal	↓	Yes	Hypercalcemia May be associated with PTH autoantibodies	Lung	Yes	No	No	Yes	No	Yes
Gazzaruso et al. (14)	28	F	Yes	Severe	Yes	Yes	UK	UK	Normal	↓	↑	UK	Yes	Hypercalcemia May be associated with PTH autoantibodies	UK	Yes	No	No	No	No	No
Deftos et al. (15)	29	M	Yes	Moderate	Yes	Yes	Yes	Yes	↑	↓	↑	↓	Yes	PTHrP production in SLE lymphoid tissue is proposed	No	Yes	No	No	Yes	No	Yes
Katchan et al. (16)	40	F	yes	Moderate	Yes	Yes	UK	No	Normal	Normal	Normal	Normal	Yes	Hypercalcemia May be associated with PTH autoantibodies	No	No	No	No	No	No	Yes
Braude et al. (17)	24	F	Yes	Severe	No	Yes	No	No	↑	↓	Normal	UK	Yes	PTHrP concentration high during hypercalcemic episodes. No origin of PTHrP found	Vessel, kidney, lung, lymph vessel	Yes	No	No	Yes	No	No
Our case	11	M	Yes	Mild	No	No	Yes	No	UK	↑	Normal	↓	No	Hypercalcemia Was attributed to high PTH and may be associated with autoantibodies to calcium sensing receptors	No	UK	Yes	No	Yes	No	No

F, female; HCA, hypercalcemic; LA, lymphadenopathy; LE, Lymphoedema; M, male; PTHrP, parathyroid-related protein; PTH, Parathyroid Hormone; RI, Renal insufficiency; UK, unknown;

* she had Jaw Neoplasms;

# *this case eventually died*.

Few articles have described the relationship between renal histopathology and the severity of hypercalcemia, especially in patients with SLE. Braude et al. (17) reported a 24-year-old woman, who was diagnosed SLE, severe hypercalcemia and renal calcium deposition. Hypercalcemia as the cause of renal calcium deposition. The relationship between hypercalcemia and immune response in renal inflammation is unclear. Kohli et al. (9) reported a 8-year-old girl, who was diagnosed SLE with class IV lupus nephritis, severe hypercalcemia, renal calcium deposits. Her kidney biopsy showed diffuse proliferative glomerulonephritis with high activity index. Deftos et al. (15) reported a 29-year-old male, who was diagnosed SLE with lupus nephritis, moderate hypercalcemia, his renal biopsy demonstrated evidence of immune complex deposition and membranous glomerulonephritis, with areas of focal proliferative change and severe interstitial nephritis. And our case was characterized by mild asymptomatic hypercalcemia. His renal biopsy was immunocomplex nephritis, mild activity, LN III. Therefore, we believe that the severity of renal histopathology may be related to the severity of hypercalcemia.

In all these cases reported in the literature, SLE with hypercalcemia is very rare, and the related mechanism is not clear. The possible pathogenesis involves elevated blood PTH level, excessive endogenous production of PTHrP or the presence of autoantibodies against PTH receptor antibodies, which mostly occur in the active phase of SLE. PTHrP may be produced by non-malignant lymphoid tissue in SLE. PTHrP has the same sequence homology with PTH, which can mediate the occurrence of hypercalcemia. Polyclonal overactivation of B lymphocytes in SLE may produce anti-PTH receptor autoantibodies, which may activate PTH receptors, bind to PTH receptors and activate PTH-mimetic effects, and inhibit the expression of PTH and PTHRP, leading to hypercalcemia. It may also produce autoantibodies to extracellular calcium-sensitive receptor (CaSR), leading to hypercalcemia. In addition, some cytokines released by active SLE patients, such as interleukin (IL)−6, IL–1, and prostaglandin E, may stimulate osteoclast bone resorption and lead to hypercalcemia. However, hypercalcemia did not occur in all active and remission SLE patients. The results showed that unknown individual factors may also contribute to the occurrence of hypercalcemia.

Our case meets American Rheumatic Society (ACR) SLE classification criteria. After excluding other common and uncommon causes of hypercalcemia, we believe that hypercalcemia may be a feature of SLE. Because of the absence of serositis and lymph node enlargement, granulomatous, SLE variant, hypercalcemic lymphadenopathy syndrome and sarcoidosis, are not supported. The presence of hypercalcemia, hypophosphatemia, and elevated levels of iPTH suggest that hypercalcemia is not secondary to the production of PTHrP and anti-PTH receptor autoantibodies, because their characteristic manifestations are low PTH levels. Because our case with hypercalcemia was in the remission stage of SLE, the probability of producing autoantibodies was low. In this patient, because immunosuppressive drugs such as glucocorticoid, MMF, and FK 506 can induce hypocalcemia, so immunosuppressive therapy does not cause hypercalcemia. Ultrasound showed hyperplasia of parathyroid gland with elevated iPTH. Considering the possibility of hypercalcemia caused by primary hyperparathyroidism, it can not entirely exclude that the production of autoantibodies to SLE is involved.

Interestingly, in our case, after routine saline infusion and furosemide diuretic treatment, blood calcium did not decrease but increase and combined with acute kidney injury stage 1 (excluding acute kidney injury caused by primary disease), suggesting that the treatment was ineffective. Our case was asymptomatic mild hypercalcemia, occasionally gastrointestinal discomfort, ineffective fluid replacement and diuresis, and then treated by oral rehydration, patient education, dietary adjustment, and avoidance of factors that may aggravate hypercalcemia (such as avoiding taking high-dose vitamin D or high-calcium foods). After treatment, serum calcium was mildly to moderately elevated. The highest level of serum calcium was 3.09 mmol/L. Studies have shown that hypercalcemia is aggravated by the loss of blood vessel content caused by insufficient intravenous fluids, which impair the renal calcium clearance rate. However, in our case, there is no dehydration and intravenous fluid infusion is sufficient, so there is no renal damage caused by loss of vascular content. We need to consider that SLE autoantibody production leads to ineffective fluid infusion and diuretic treatment. Although our case was in remission stage, it was not excluded that SLE produces autoantibodies to extracellular calcium-sensitive receptors, which leaded to elevate levels of hypercalcemia and PTH, so it was ineffective for rehydration and diuretic therapy. Considering that the pathogenesis of SLE complicated with hypercalcemia complicates the response of drug treatment, the medical treatment of SLE-related hypercalcemia is necessary. Clinicians should be more cautious in choosing treatment options and monitoring relevant indicators.

## Data Availability Statement

All datasets generated for this study are included in the article/supplementary material.

## Ethics Statement

This study was approved by the ethics committee of the First Affiliated Hospital, Sun Yat-sen University. Signed informed consent was obtained from the parents of this patient.

## Author Contributions

XJ conceptualized and designed the study, reviewed, and revised the manuscript. LX carried out the initial analyses and drafted the initial manuscript. JZ, RP, and YM were responsible for the treatment of the patient. LX and RP coordinated and supervised the data collection. ZY and XJ critically reviewed the manuscript. All authors read and approved the final manuscript.

### Conflict of Interest

The authors declare that the research was conducted in the absence of any commercial or financial relationships that could be construed as a potential conflict of interest.

## Abbreviations

BUN: urea nitrogen
CCR: creatinine clearance rate
CR: creatinine
CT: computed tomography
F: female
FK 506: tacrolimus
HCA: hypercalcemic
LA: lymphadenopathy
LE: Lymphoedema
LN: Lupus nephritis
M: male
MMF: Mycophenolate mofetil
PTH: parathyroid hormone
PTHrP: parathyroid-related protein
RI: Renal insufficiency
SLE: systemic lupus erythematosus
SLEDAI: systemic lupus erythematosus disease activity
SPECT: single-photon emission computed tomography
UK: unknown.
